# A MAM7 Peptide-Based Inhibitor of *Staphylococcus aureus* Adhesion Does Not Interfere with *In Vitro* Host Cell Function 

**DOI:** 10.1371/journal.pone.0081216

**Published:** 2013-11-12

**Authors:** Catherine Alice Hawley, Charlie Anne Watson, Kim Orth, Anne Marie Krachler

**Affiliations:** 1 Institute of Microbiology and Infection, University of Birmingham, Birmingham, United Kingdom; 2 Department of Molecular Biology, University of Texas Southwestern Medical Center, Dallas, Texas, United States of America; University of Strathclyde, United Kingdom

## Abstract

Adhesion inhibitors that block the attachment of pathogens to host tissues may be used synergistically with or as an alternative to antibiotics. The wide-spread bacterial adhesin Multivalent Adhesion Molecule (MAM) 7 has recently emerged as a candidate molecule for a broad-spectrum adhesion inhibitor which may be used to prevent bacterial colonization of wounds. Here we have tested if the antibacterial properties of a MAM-based inhibitor could be used to competitively inhibit adhesion of methicillin-resistant *Staphylococcus aureus* (MRSA) to host cells. Additionally, we analyzed its effect on host cellular functions linked to the host receptor fibronectin, such as migration, adhesion and matrix formation *in vitro*, to evaluate potential side effects prior to advancing our studies to *in vivo* infection models. As controls, we used inhibitors based on well-characterized bacterial adhesin-derived peptides from F1 and FnBPA, which are known to affect host cellular functions. Inhibitors based on F1 or FnBPA blocked MRSA attachment but at the same time abrogated important cellular functions. A MAM7-based inhibitor did not interfere with host cell function while showing good efficacy against MRSA adhesion in a tissue culture model. These observations provide a possible candidate for a bacterial adhesion inhibitor that does not cause adverse effects on host cells while preventing bacterial infection.

## Introduction

 Wound infections are increasingly challenging to treat due to a rise in multidrug-resistant (MDR) bacterial isolates. While MDR Gram-negative bacteria such as *Klebsiella pneumoniae* and *Pseudomonas aeruginosa* increasingly contribute to the profile of wound infections seen in the clinic, Gram-positives and above all methicillin-resistant *Staphylococcus aureus* (MRSA) remain a major cause of morbidity and mortality in wounded patients [1,2]. As an alternative approach to antimicrobial treatment of wound infections, we are studying the potential of targeting bacterial-host interactions using adhesion inhibitors. Prevention of bacterial attachment to host tissues abrogates subsequent processes facilitating infection, such as type III secretion system (T3SS)-mediated effector injection into host cells or cellular invasion, making this a promising strategy for management of bacterial infections [3].

*S. aureus* employ an array of adhesins to achieve host cell attachment and invasion and exploits fibronectin as a key receptor for cell attachment and invasion [4-6]. Attempts have been made to utilize peptides derived from fibronectin-binding proteins (FnBPs) as adhesion inhibitors[6,7]. For example, a recombinant fragment of the *S. aureus* adhesin fibronectin-binding protein A (FnBPA) diminished staphylococcal abscess formation in a guinea pig model of wound infection and had a synergistic effect on conventional antibiotic treatment [7]. However, the competitive properties of these molecules are based on their ability to bind to the host receptor fibronectin with high affinity. Since fibronectin is tightly involved in a range of cellular processes prerequisite to wound healing, such as cellular proliferation, adhesion, migration and matrix formation [8], this caused undesired side-effects on host cellular functions [9,10].

We have recently identified a novel family of bacterial adhesins, termed Multivalent Adhesion Molecules (MAMs). MAMs are involved in initial bacterial attachment to host cells and MAM homologs are found in many Gram-negative pathogens [11]. MAMs are outer membrane proteins consisting of tandem arrays of six to seven mammalian cell entry (mce) domains. The mce domains mediate attachment to host tissues by high affinity interaction with the host membrane lipid phosphatidic acid (PA) and utilize fibronectin as a co-receptor [12]. Since MAM homologs are present in many bacterial species, the use of MAM-based inhibitors might be an approach allowing prophylaxis and eventually treatment of a broad spectrum of infections [13]. We have successfully used inhibitors based on *Vibrio parahaemolyticus* MAM7 to prevent infections caused by enteric pathogens in tissue culture models, and more recently we demonstrated that this approach can be extended to MDR Gram-negative isolates causing wound infections [14]. 

**Figure 1:**
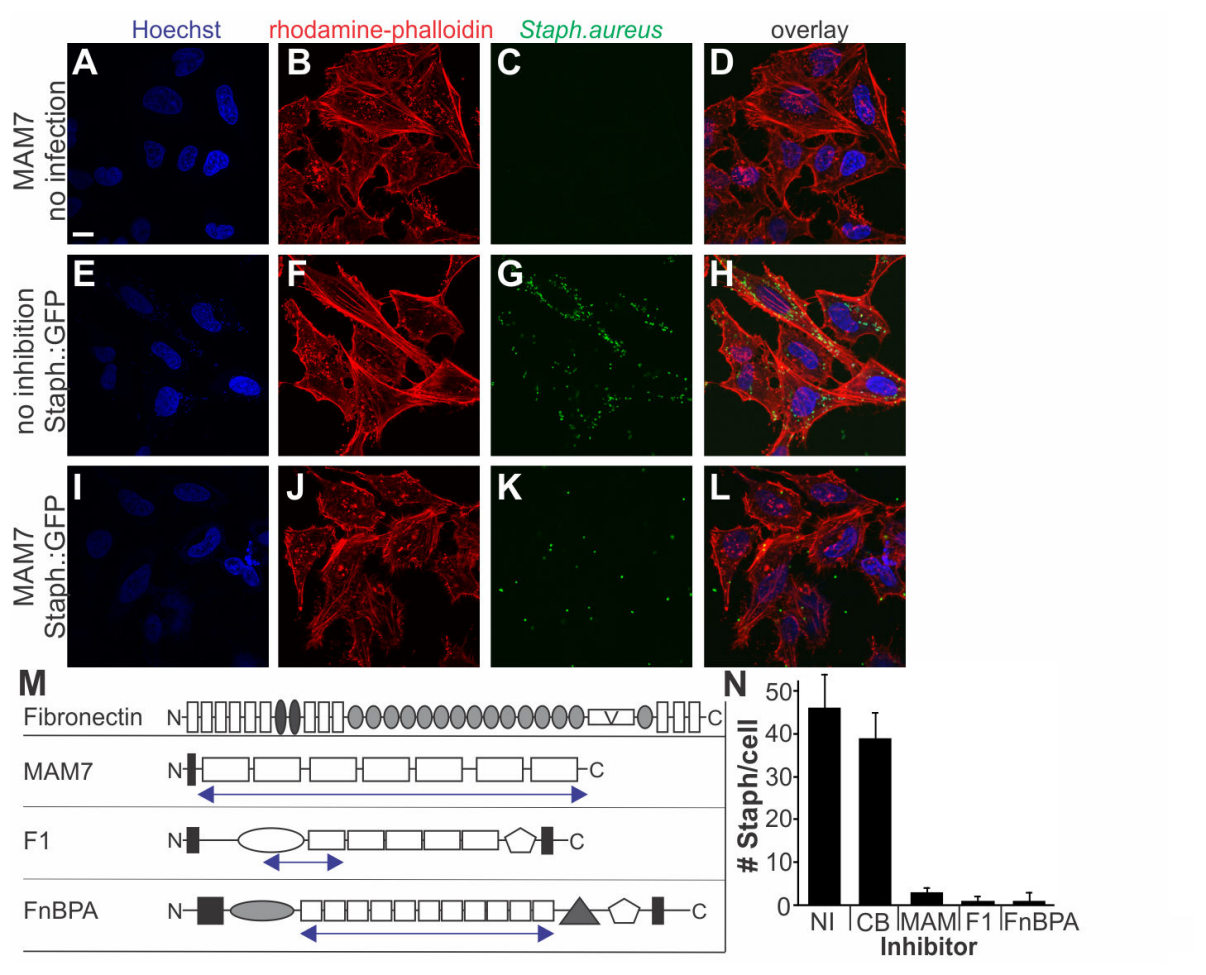
Adhesion inhibitors protect host cells from MRSA infection. GST or peptides derived from MAM7, F1 and FnBPA (blue arrows) were purified and immobilized on latex beads for inhibition experiments (M). HeLa cells incubated with MAM7-based inhibitor (A-D and I-L) and infected with *S*. *aureus* USA300:GFP (E-L). DNA (blue, A, E, L), F-actin (red, B, F, J) and *S*. *aureus* USA300:GFP (green, C, G, K) were visualized by fluorescence microscopy. Scale bar, 20 μm. *S*. *aureus* were counted after infection in the presence of no inhibitor, NI or immobilized GST, CB, MAM7, F1 or FnBPA-peptide inhibitors (N). Results are means ± standard deviation from analyzing 15 randomly chosen images per sample, from experiments done in triplicate.

Since the binding site in fibronectin recognized by MAM7 is also recognized by *S. aureus* FnBPA, we set out to test if the antibacterial properties of MAM7 could be extended to competitively inhibit *S. aureus* adhesion to host cells. Additionally, we analyzed the effects of a MAM7-based adhesion inhibitor on host cellular responses *in vitro*, to evaluate potential side effects prior to advancing our studies to animal infection models. Along with a MAM7-based inhibitor, we included in our study two other well-characterized adhesins utilizing fibronectin as host receptor (Figure 1): The surface protein F1 is a major adhesin of *Streptococcus pyogenes*, a Gram-positive bacterium and an opportunistic pathogen causing a spectrum of diseases, ranging from skin infections and strep throat to toxic shock syndrome. F1 mediates tissue colonization and invasion by binding to the N-terminal region of Fn with nanomolar affinity [15,16]. The major fibronectin-binding site in F1 is located to the functional upstream domain (FUD), which encompasses the 43 residues of the F1 upstream nonrepetitive domain and six residues of the first of five repeat domains (Figure 1M), [9,17]. Previous studies suggested that FUD binding to tissue may interfere with cellular functions such as vascular remodeling [10].The second control peptide we used was derived from *S. aureus* FnBPA, which had previously been investigated as adhesion inhibitor [7]. FnBPA mediates bacterial attachment and invasion of a variety of cell types by attaching to the N-terminal region of fibronectin in a modular fashion, using a tandem β-zipper mechanism [18-20]. FnBPA contains eleven fibronectin-binding repeats (FnBRs) arranged in tandem, and the binding affinity of individual repeats ranges from 1nM to 3μM (Figure 1M), [21]. 

Our studies demonstrate that adhesion inhibitors based on peptides derived from adhesins F1 and FnBPA efficiently block bacterial adhesion but interfere with cellular processes promoting wound healing, as was previously described. In contrast, a MAM7-derived peptide is an effective adhesion inhibitor but does not cause undesired effects on host cells *in vitro*. 

## Materials and Methods

### Cloning, expression and purification of bacterial proteins

The MAM7-derived peptide contained residues 45-882 of MAM7 (VP1611) and an N-terminal GST-tag. The F1-derived peptide corresponds to F1 functional upstream domain (FUD), as described previously [9]. Cloning, expression and purification of MAM7 and F1 FUD peptides have been described elsewhere [9,11]. The FnBPA-derived peptide contained fibronectin-binding repeats 1-11 (FnBR1-11) according to domain boundaries as described previously [21]. The construct containing FnBPA FnBR1-11 was amplified from *S. aureus* USA300 genomic DNA and cloned into pGEX-TEV using BamHI and NotI sites. Protein was expressed in *E. coli* BL21 and purified on glutathione sepharose as previously described for individual FnBRs [21]. 

### *Staphylococcus aureus* adhesion to host cells

GST, MAM7, F1 or FnBPA derived peptides were immobilized on latex beads as previously described [13]. Bead-immobilized inhibitors in culture medium were added to plates containing HeLa cells to give a final concentration of 500 nM immobilized peptides and incubated for one hour. Medium was then replaced with medium, containing GFP-expressing *S. aureus* USA300 (gift from V. Torres lab) at a multiplicity of infection of 10 and incubated for four hours. Cells were washed, fixed with 3.2% formaldehyde, permeabilized with 0.5% Triton X-100 in PBS and stained for F-actin using rhodamine-phalloidin and for nucleic acid using Hoechst 33342 as previously described [11]. Samples were imaged using a fluorescence microscope and bacterial counts per cell were determined by counting at least 15 images per sample. Images were processed using Image J and Corel X5. 

### Analysis of MAM-inhibitor efficacy against enteric infections

HeLa cells were infected with *V. parahaemolyticus, V. cholerae, Yersinia pseudotuberculosis* or enteropathogenic *E. coli* (EPEC) and cytotoxicity or actin pedestals/cell (for EPEC) measured as previously described [11]. 

### Scratch assays

HaCaT [22] and human dermal fibroblast (HDF) cell lines [23] were a gift from the Hotchin lab (Univ. of Birmingham). HaCaT, HDF and HeLa (ATCC number CRM-CCL-2) cells were cultured in Dulbecco’s MEM supplemented with 10% heat-inactivated fetal bovine serum (FBS) and 50 μg/ml penicillin and streptomycin at 37°C with 5% CO_2_. Cells were grown to a confluent monolayer which was scratched with a pipette tip and washed with DMEM once. Cells were incubated with fresh DMEM alone or containing 500 nM immobilized GST, MAM7, F1 or FnBPA derived peptide and DIC images were taken at t=0, 6, 12, 18 and 24 hours after scratching. Cell migration into the cell-free gap and gap closure was evaluated by measuring the gap between the two migrating fronts using Image J and normalizing to the gap width at t=24 hours (100% closure).

### Analysis of peptide integrity

500 nM MAM7, F1 or FnBPA derived peptides in culture medium was added to monolayers of fibroblasts and incubated at 37°C. Supernatant samples were taken following 0, 6, 12, 18 and 24 hours of incubation and protein precipitated using TCA precipitation. Samples were separated by SDS-PAGE and amount of intact peptide was quantified using densitometry and normalization to the peptide band from the sample taken at 0 hours.

### Cell viability assays

Cells were seeded into 96-well plates at a density of 1.5·10^5^ cells/ml one day prior to the experiment. Cultured cells were incubated with DMEM alone or medium supplemented with 500 nM immobilized GST, MAM7, F1 or FnBPA peptides. Cell viability after 4 hours and 24 hours of incubation was determined by measuring lactate dehydrogenase (LDH) activity in cell-free supernatants using an LDH Cytotoxicity Detection Kit (Clontech) according to the manufacturer’s instructions. Treatment with 1 μM staurosporine was used as a positive control and results were normalized to total lysis controls (cells lysed with 0.5 % Triton X-100, resulting in 100% cell death). 

### Cell proliferation assays

Cells were seeded into 96-well plates at a density of 1.5·10^5^ cells/ml one day prior to the experiment. Proliferation of cells grown in the absence or presence of 500 nM immobilized GST, MAM7, F1 or FnBPA derived peptide for 24 hours was measured using the CyQUANT NF Cell Proliferation Kit (Life Technologies) according to the manufacturer’s instructions. Cells grown in medium alone were used as a positive control (100% proliferation) and for normalization. 

### Cell adhesion assays

Cell suspensions were prepared either in medium alone or medium containing immobilized GST, MAM7, F1 or FnBPA derived peptides, to give a final concentration of 1.5·10^5^ cells/ml and 500 nM peptide. Experiments were carried out both in the presence or absence of 10% fetal bovine serum. Cells were seeded into 24-well plates and left in the incubator to attach to the culture vessel for 2 or 12 hours, respectively. Medium was removed and wells were washed three times with PBS. Attached cells were removed using trypsin-EDTA and counted using a hemocytometer. Attachment was normalized to medium only control samples (100% attachment). 

### Matrix formation assays

Matrix formation was both visualized and quantified using the incorporation of rhodamine-labelled fibronectin as reporter, as described previously [9,24]. Briefly, fibronectin from human plasma (Sigma) was prepared at 1 mg/ml in PBS and labeled with a 10-fold molar excess of NHS-rhodamine for one hour at 22°C. Excess label was removed using desalting columns. Fibroblasts were seeded at 2·10^5^ cells/ml onto 6-wells containing coverslips (for visualization) or 96-well plates (for quantification) in the presence or absence of 500 nM immobilized GST, MAM7, F1 or FnBPA derived peptides. After one hour, rhodamine-fibronectin was added to a final concentration of 20 nM and cells were incubated 12 or 24 hours, respectively. For visualization, cells were formaldehyde-fixed, mounted and imaged using a fluorescence microscope fitted with a 60x oil immersion lens. Images were processed using Image J and Corel X5. Incorporation of labeled fibronectin was quantified after 12 and 24 hours, respectively, as described previously [24]. Controls were visualized immediately to control for background fluorescence. Matrix assembly was expressed as percentage and normalized to medium control samples (100% assembly). 

## Results

### Synthetic adhesion inhibitors based on MAM7, F1 and FnBPA - derived peptides protect host cells from MRSA adhesion

It was previously shown that prophylactic treatment with *V. parahaemolyticus* MAM7-based adhesion inhibitors protects host cells against subsequent infection with Gram-negative pathogens in tissue culture models of infection. This is based on competitive binding of the inhibitor to host cell receptors, preventing pathogens from attaching [11]. Following our previous work on MAM7-based adhesion inhibition of enteric pathogens, we attempted to use recombinant purified MAM7 peptide, as well as a shorter peptide (MAM7 mce1 domain) as inhibitors of infection with *V. parahaemolyticus, V. cholerae, Yersinia pseudotuberculosis* or enteropathogenic *E. coli* (EPEC) and found the efficacy of these to be significantly lowered compared to that of MAM7-expressing non-pathogenic bacteria. In contrast, when the longer (MAM7) peptide was coupled to a polymer bead comparable in size to the bacteria used, the inhibitory effect was fully restored (Figure S1). Consequently, further adhesion inhibition studies were carried out using bead-coupled MAM7 peptide. 

We tested whether this synthetic MAM7-based inhibitor could also be used to protect cells against *Staphylococcus aureus*, a major causative agent of wound infections. We incubated HeLa cells with GFP-expressing methicillin-resistant *S. aureus* USA300, either with our without prior treatment with bead-immobilized MAM7 peptide. MAM7-treated cells showed significantly diminished *S. aureus* adhesion compared to untreated controls (Figure 1). 

We additionally used purified peptides derived from *Streptococcus pyogenes* F1 (FUD, subsequently referred to as F1 peptide) and *S. aureus* FnBPA (FnBR1-11, subsequently referred to as FnBPA peptide) to see whether these proteins could efficiently inhibit cellular attachment of *S. aureus* (Figure 1M). Both F1 and FnBPA peptides were immobilized to beads and used to treat cells prior to incubation with *S. aureus*. While *S. aureus* incubation of untreated host cells lead to bacterial counts of approximately 46 bacteria per cell, pre-treatment with either MAM7, F1 or FnBPA-based peptide inhibitors lead to a decrease in bacterial counts to between one and three bacteria per cell, while treatment with GST-immobilized control beads did not significantly affect the number of host-associated bacteria (Figure 1N). 

### F1 and FnBPA-based inhibitors, but not a MAM7-based inhibitor, delay wound healing in scratch assays

**Figure 2:**
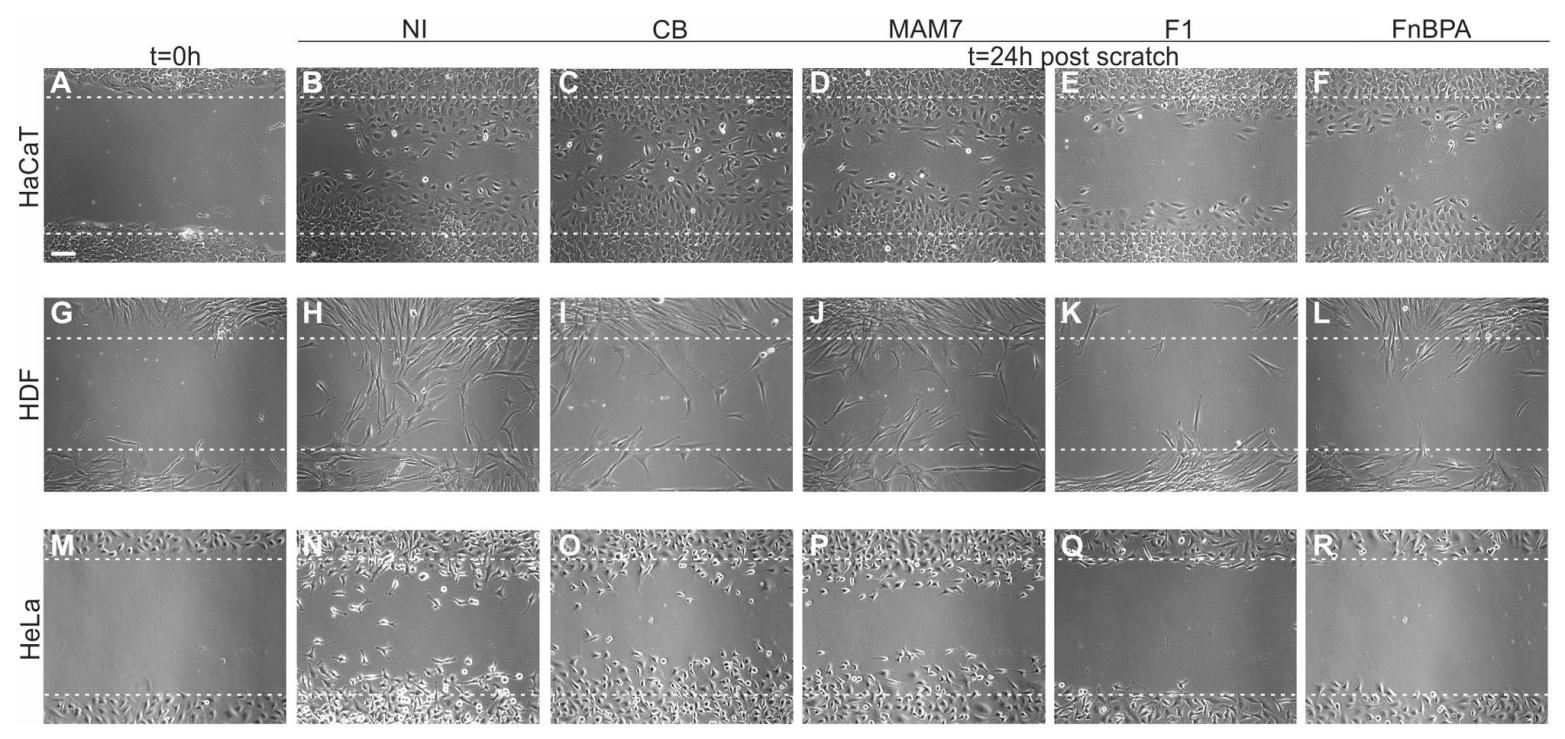
Effects of adhesion inhibitors on cellular migration. Confluent layers of HaCaT (top), HDF (middle) or HeLa cells (bottom) were scratched and imaged immediately (t=0h, A, G, M) or following 24 hours of incubation in medium alone (NI, B, H, N) or medium containing 500 nM immobilized GST (CB, C, I, O), MAM7 (D, J, P), F1 (E, K, Q) or FnBPA peptides (F, L, R). Scale bar, 50 μm. Images are representative of a set of three experiments done in triplicate, with three frames taken per replicate. Images were processed using Image J and Corel X5.

**Figure 3:**
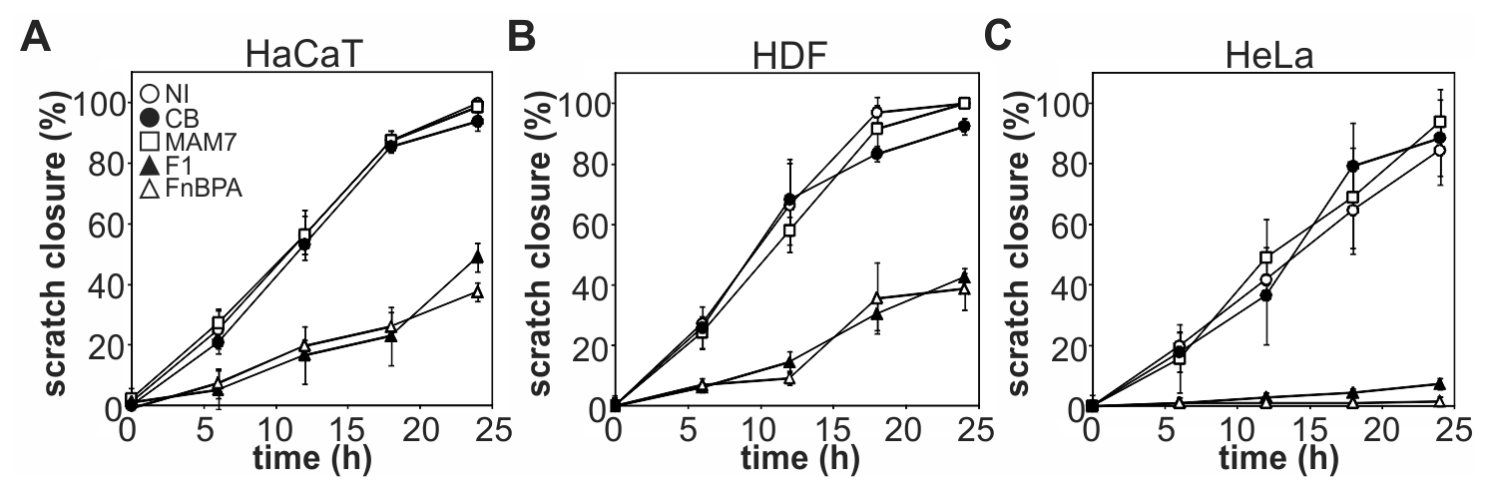
Effects of adhesion inhibitors on the rate of scratch closure. Experiments were performed as depicted in Figure 2 and scratch closure on confluent layers of HaCaT (A), HDF (B) or HeLa cells (C) was determined at t=0, 6, 12, 18 and 24 hours post scratch. Scratch width at 24 hours post scratch was defined as 100 % closure. Results are means ± standard deviation from one of a set of three experiments done in triplicate wells, with three frames taken per well. NI, no inhibitor present (open circles); CB, GST-immobilized control beads (black circles), MAM7, bead-immobilized MAM7 peptide (open squares); F1, bead-immobilized FUD peptide (closed triangles); FnBPA, bead-immobilized FnBPA (open triangles).

Wound closure is a complex process, but scratch assays on monolayers are often used to mimic wound healing *in vitro* and to determine the influence of exogenous molecules on the healing process in a simple model system. To test whether the presence of adhesion inhibitors would influence wound healing *in vitro*, we performed scratch assays and imaged scratched monolayers of HaCaT, HDF and HeLa cells either immediately or following a 24 hour period in the presence or absence of 500 nM immobilized GST, MAM7, F1 or FnBPA derived peptide (Figure 2). In the absence of inhibitor, scratched layers of HaCaT and HDF cells fully closed the scratch and HeLa cells, which migrated slighty slower, almost closed the gap within 24 hours (Figure 2N). Similar results were obtained in the presence of control beads (Figure 2C, I, O) or MAM7 inhibitor (Figure 2D, J, P). In the presence of either F1 or FnBPA inhibitors, scratch closure was compromised with all three cell lines. While for HaCaT and HDF cells scratch closure was decreased by approximately 50 % in the presence of either F1 or FnBPA inhibitors, healing was almost completely inhibited in HeLa cells (Figure 2E, F, K, L, Q, R). The duration of all experiments was limited to 24 hours and peptide integrity was maintained over that period (Figure S2). To quantify the inhibitory effects of bacterial peptides on scratch closure, we determined the progress in scratch closure after 0, 6, 12, 18 and 24 hours of incubation (Figure 3). The results were in agreement with those obtained from initial images: While control beads or the MAM7 inhibitor had no significant effect on scratch closure, both F1 and FnBPA inhibitors delayed healing to a similar extent, with the inhibitory effect being most severe on HeLa cells (Figure 3C). 

### Inadequate wound healing is not due to increased cell death or decreased proliferation

**Figure 4:**
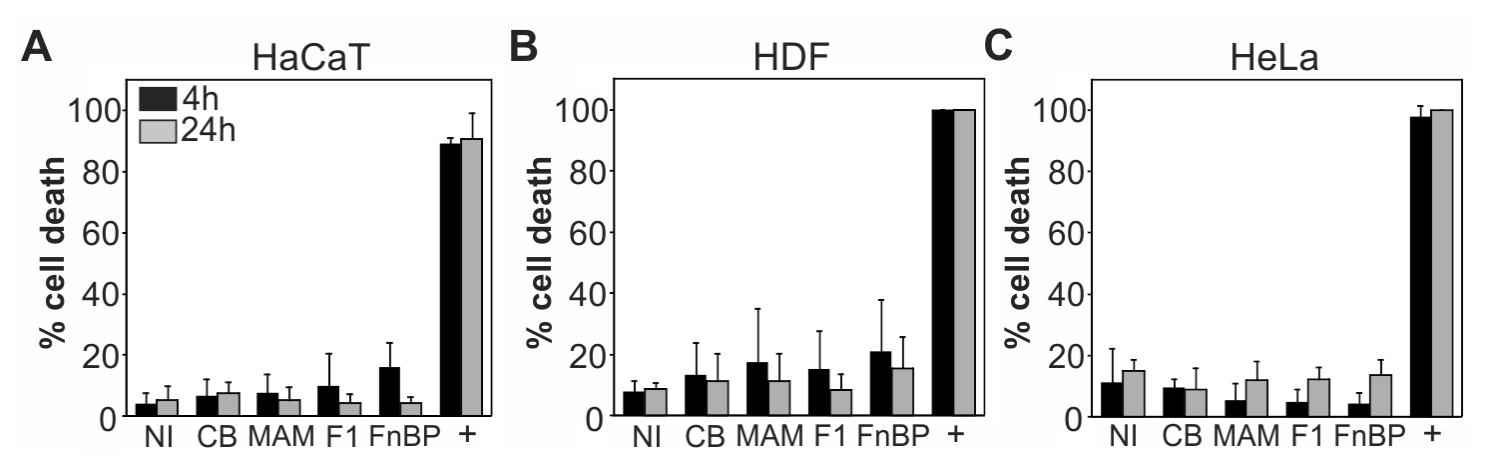
Adhesion inhibitors do not affect host cell survival. Cell death of HaCaT (A), HDF (B) or HeLa cells (C) was measured using lactate dehydrogenase release assays following 4 hours (black) or 24 hours (gray) of incubation in growth medium alone (NI) or medium containing 500 nM immobilized GST (CB), MAM7, F1 or FnBPA peptide, or 1μM staurosporine (+ control). Results are means ± standard deviation from one of a set of three experiments done in triplicate. According to student t-test, p<0.01 for all samples compared to positive control.

**Figure 5:**
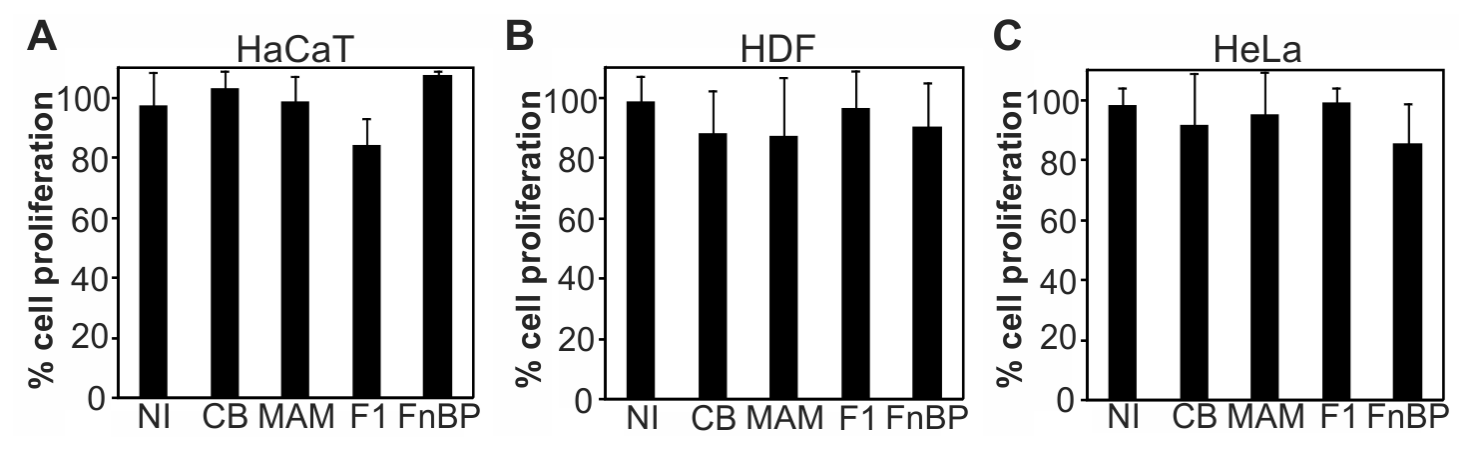
Adhesion inhibitors do not affect host cell proliferation. Proliferation of HaCaT (A), HDF (B) or HeLa cells (C) was measured using a CyQUANT NF proliferation assay in medium alone (NI) or medium containing 500 nM immobilized GST (CB), MAM7, F1 or FnBPA peptides. Results are means ± standard deviation from one of a set of three experiments done in triplicate.

Decreased rates of scratch closure could be due to increased cell death or decreased proliferation of cells exposed to adhesion inhibitors. To test whether the inhibitors increased cell death, HaCaT, HDF and HeLa cells were grown in the presence or absence of GST, MAM7, F1 or FnBPA inhibitors for 4 hours or 24 hours, respectively, followed by measurements of lactate dehydrogenase (LDH) activity in the cell supernatant. Cell death causes an increase in membrane permeability, which results in LDH release from cells into the supernatant. As a total lysis control (100% cell death), cells were lysed by addition of 0.5% Triton X-100. Neither of the four immobilized peptides caused a significant increase in LDH release from cells and thus cell death (Figure 4). Addition of 1 μM staurosporine (positive control), which causes cells to undergo apoptotic cell death, killed close to 100% of cells in all three cell lines within the same timeframe. Next, we tested if attachment of adhesion inhibitors to host cells would cause a decrease in proliferation rates. Cell lines were grown either with or without prior addition of 500 nM GST, MAM7, F1 or FnBPA inhibitors to the growth medium and proliferation was determined after 24 hours using the CyQUANT NF Cell Proliferation Kit. None of the four peptides caused a significant change in cell proliferation compared to cells grown in medium alone. Similar results were obtained for all three cell lines tested (Figure 5). 

### Adhesion inhibitors can cause decreased adhesion of host cells

**Figure 6:**
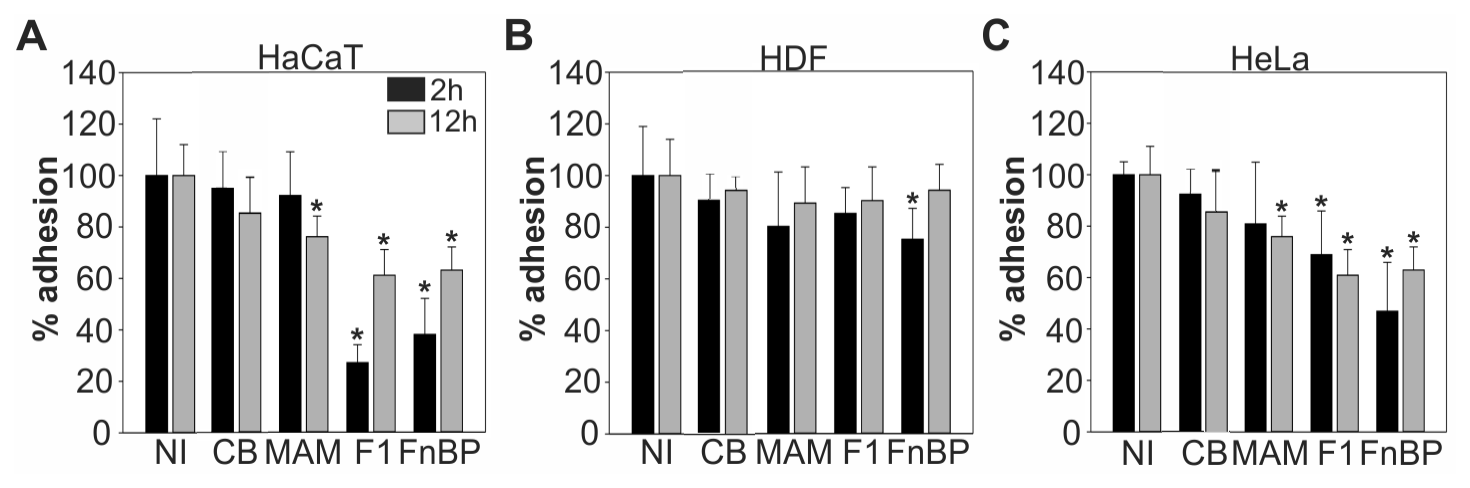
Effect adhesion inhibitors on host cellular adhesion in the presence of serum. Adhesion of HaCaT (A), HDF (B) or HeLa cells (C) to culture vessels was measured following 2 hours (black) or 12 hours (grey) of incubation in medium containing 10 % FBS alone (NI) or medium containing 500 nM immobilized GST (CB), MAM7, F1 or FnBPA peptide. Results are means ± standard deviation from one of a set of three experiments done in triplicate. Values significantly different from the control (p<0.01 according to student t-test) are indicated (*).

Cellular matrix adhesion is a prerequisite for migration and thus wound healing. To test whether adhesion inhibitors could influence the cells’ ability to adhere to a matrix, trypsin-treated cells were washed and then seeded in culture vessels either in the presence or absence of 500 nM GST, MAM7, F1 or FnBPA based inhibitors, and the number of adherent cells was determined using a hemocytometer, following either 2 hours or 12 hours of incubation. Cells in medium alone were used as a positive control (NI, 100% adherence). Under serum-free conditions, the adherence of all three cell types was significantly decreased in the presence of either F1 or FnBPA based inhibitors at both time points, while no measurable effect was observed with control beads (Figure S3). The MAM7 inhibitor caused a slight decrease in adherence of HaCaT and HeLa cells after 2 hours, but no significant differences were observed after 12 hours of incubation or on HDF cells. To better mimic *in vivo* conditions, the experiments were repeated in medium containing 10% serum. Under these conditions, the inhibitors’ ability to impair cellular adhesion to the substrate was generally less pronounced. No significant differences in HDF adherence were seen in the presence or absence of inhibitors, with the exception of FnBPA after 2 hours (Figure 6B). However, a decrease in adherence of HaCaT and HeLa cells in the presence of serum at both time points was observed for cells plated with F1 and FnBPA inhibitors, (Figure 6A, C). No significant difference was observed with control beads or MAM7 inhibitor at 2 hours with minor inhibition of adherence observed with MAM7 inhibitor at 12 hours (Figure 6A, C). 

### F1 and FnBPA-based inhibitors, but not a MAM7-based inhibitor, impair extracellular matrix formation

**Figure 7:**
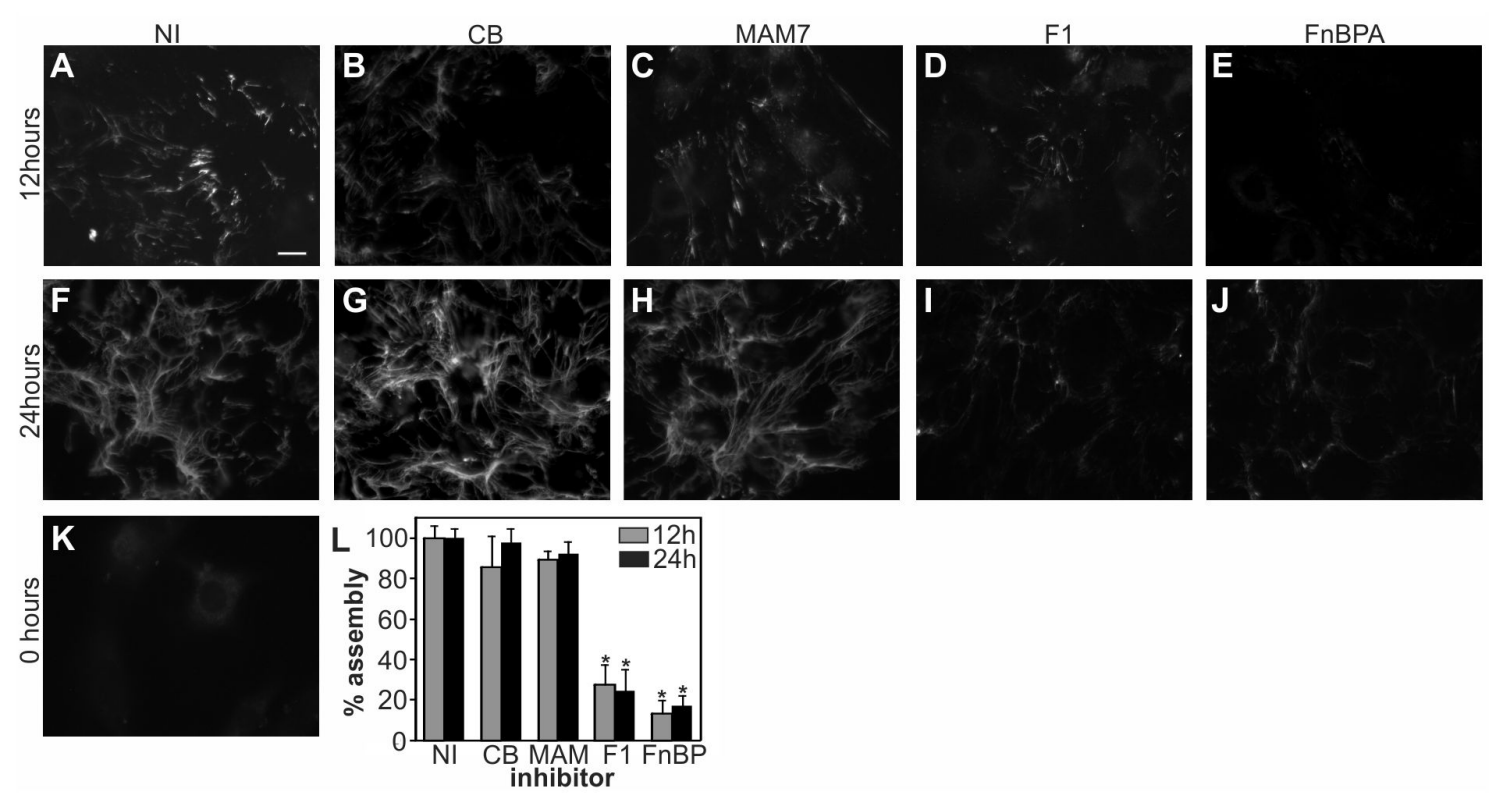
Influence of adhesion inhibitors on *in* *vitro* matrix formation. Incorporation of rhodamine-labelled fibronectin into the extracellular matrix of fibroblasts was visualized by fluorescence microscopy, after 12 or 24 hours of incubation in medium alone (NI, A, F) or medium containing immobilized GST (CB, B, G), MAM7 (C, H), F1 (D, I) or FnBPA (E, J) derived peptides. Background fluorescence of unlabelled cells at t=0 hours (K). Scale bar, 10 μm. Images are representative of a set of three experiments, with at least three frames taken per replicate. Incorporation of rhodamine-fibronectin into the extracellular matrix in medium alone (NI) or medium containing immobilized GST (CB), MAM7, F1 or FnBPA derived peptides was quantified after 12 hours (grey) and 24 hours (black) incubation using fluorescence plate assays (L). Values significantly different from the control (p<0.01 according to student t-test) are indicated (*).

Adhesion is influenced by the cells’ ability to form an extracellular matrix, which acts as a natural substratum for cell attachment. To test if adhesion inhibitors would influence extracellular matrix formation, HDF cells were grown in the presence or absence of 500 nM GST, MAM7, F1 or FnBPA inhibitors and matrix formation was evaluated visually and quantitative using fluorescence plate assays. Exogenously added rhodamine-labeled fibronectin, which gets incorporated into the cells’ endogenous matrix, was used as a probe for matrix formation. Cells were visualized by fluorescence microscopy following 12 and 24 hours of incubation, respectively (Figure 7). In medium alone (NI), fibronectin fibrillogenesis was apparent after 12 hours, and by 24 hours a dense meshwork of long fibers had formed (Figure 7A, F). Similar results were obtained in the presence of control beads or MAM7 inhibitor (Figure 7b,c,g,h). In contrast, in the presence of either F1 or FnBPA inhibitors, fibronectin fibers were less dense, shorter and thinner than in the control (Figure 7D, E, I, J). Matrix formation was quantified by measuring the amount of rhodamine-fibronectin incorporated into endogenous fibronectin fibrils in the presence or absence of inhibitors after 12 and 24 hours, respectively. No significant decrease in fluorescence was detected between untreated wells and wells containing either control beads or MAM7 inhibitor at either time point (Figure 7L). However, the presence of either F1 or FnBPA inhibitors lead to a decrease of fluorescence levels by approximately 80 and 90%, respectively, reflecting a decrease in matrix formation after both 12 and 24 hours (Figure 7L). 

## Discussion

Many bacterial pathogens rely on fibronectin as a surface receptor for host attachment, so competitive adhesion inhibitors based on fibronectin-binding domains derived from bacterial adhesins have large potential for future use in the management of bacterial infections. However, fibronectin is a key molecule mediating a range of cellular processes necessary for wound healing, such as cell proliferation, adhesion, migration and matrix formation and thus fibronectin binding by adhesins or adhesin-derived peptides can interfere with these processes and potentially lead to undesired side-effects such as delays in wound healing and aberrant vascular remodeling [10]. To test for potential side-effects which might be caused by topical treatment of wounds, we utilized cultured host cells to test an adhesion inhibitor based on a peptide derived from *V. parahaemolyticus* MAM7 for its effect on *S. aureus* attachment as well as host cellular functions *in vitro*. As controls, we included two adhesion inhibitors based on fibronectin-binding peptides derived from *S. pyogenes* F1 and *S. aureus* FnBPA, respectively, in our analysis.

We found that all three peptides, when immobilized on beads, efficiently inhibited adhesion of the methicillin-resistant *S. aureus* strain USA300 to host cells at nanomolar concentrations, demonstrating the potential benefit of such inhibitors for the treatment of MDR infections. As a first step towards further development of the MAM7-based inhibitor, we analyzed its effect and the effect of the two control inhibitors on host cellular functions. Fibroblasts and keratinocytes are cell types that play important roles in wound healing. Since we aim to develop MAM7-based adhesion inhibitors for topical treatment of wounds, we analyzed host cellular function in HaCaT and HDF cell lines. As additional control, we tested the effects on HeLa cells, an unrelated but well characterized cell type not involved in cutaneous repair.

Both F1 and FnBPA-based inhibitors, although efficient in preventing bacterial adhesion, caused a delay in wound healing in tissue culture models (Figures 2, 3). This inhibitory effect was not due to changes in cell viability or proliferation caused by the peptides. However, both F1 and FnBPA inhibitors impaired extracellular matrix formation and cellular adhesion. This is in agreement with previous studies, which reported that the F1-derived peptide FUD impairs matrix formation and, dependent on the peptide concentration, may impair cellular adhesion [9]. Deposition of soluble fibronectin into fibrils is one of the initial steps in extracellular matrix formation and fibronectin fibrils act as a basis for the deposition of other matrix proteins, such as collagen, fibrinogen and fibulin [25-27]. Accumulation of fibronectin is dependent on the interaction of soluble fibronectin and adherent cells, which causes a conformational change in fibronectin that is necessary for fibrillogenesis [28]. High-affinity bacterial fibronectin-binding peptides, but not the lower affinity ligand MAM7, interfere with both soluble and deposited fibronectin assembly, thus blocking one of the fundamental steps in the healing process. Formation of extracellular matrix in turn is a prerequisite for cellular adhesion and this explains why inhibition of matrix formation by F1 and FnBPA-based inhibitors resulted in a significant decrease of cellular adhesion [29]. Taken together, impairment of these two steps is sufficient to cause delayed migration and thus wound healing and explains the phenotype observed in *in vitro* wound healing assays. 

In conclusion, our studies suggest there is a trade-off between high affinity binding to fibronectin and inhibition of wound healing, which likely precludes most bacterial fibronectin-binding peptides from being used as anti-adhesion compounds. Further studies on adhesin-based inhibitors should take this into account and carefully evaluate the impact of such inhibitors on cellular function. In contrast to F1 and FnBPA, MAM7 uses the host cell lipid phosphatidic acid as a main receptor and its interaction with this lipid species is the basis for high affinity binding of the adhesin to host cells. Fibronectin acts as a co-receptor to increase the on-rate of binding but its interaction with MAM7 is of relatively low affinity [12]. This synergistic mode of host cell engagement means that MAM7 based inhibitors stably attach to host cells and are able to competitively inhibit binding of MRSA, which uses fibronectin as a receptor, without abrogating fibronectin-mediated host cellular functions. Thus, its mechanism of receptor binding suggests MAM7 is a good candidate molecule for the development of anti-adhesion lead compounds and we will take the presented results as a basis to progress the development of these compounds towards testing them in an *in vivo* wound healing model. 

## Supporting Information

Figure S1 **Comparison of different MAM7-based inhibitors for their efficacy against enteric pathogens.** Following treatment with MAM-based adhesion inhibitors, Hela cells were infected with *V. parahaemolyticus* (**A**), *V. cholera* (**B**), *Y. pseudotuberculosis* (**C**) or EPEC (**D**), as previously described [11]. Inhibitors consisted either of MAM7 peptide (7) or MAM7 mce1 domain (1) expressed on the surface of *E*. *coli* BL21 (bact.), purified recombinant protein (prot) or bead-coupled protein (bead). As control, cells were treated with BL21 expressing MAM7ΔN_1-44_ (not surface exposed), GST-tag only (prot C) or bead-coupled GST-tag (bead C). (+) infected cells without prior treatment; (-) uninfected cells; LDH release was measured and drawn as % cell lysis (normalized to 100% = detergent lysed cells). For EPEC, pedestals per cell were counted as previously described [11]. Results are means ± standard deviation from experiments done in triplicate.
(TIF)

Figure S2 **Integrity of anti-adhesion peptide inhibitors over time.** The integrity of MAM7, F1 or FnBPA peptides co-incubated with fibroblast monolayers was determined using SDS-PAGE and densitometry. Values are expressed as % integrity and results are means ± standard error from three replicates.
(TIF)

Figure S3 **Effect of adhesion inhibitors on host cellular adhesion in the absence of serum.** Adhesion of HaCaT (a), HDF (b) or HeLa cells (c) to culture vessels was measured following 2 hours (black) or 12 hours (grey) of incubation in serum-free medium alone (no inhibitor, NI) or medium containing 500 nM bead-immobilized GST (CB), MAM7, F1 or FnBPA peptide. Results are means ± standard deviation from one of a set of three experiments done in triplicate. Values significantly different from the control (p<0.01 according to student t-test) are indicated (*).
(TIF)
