# Supplementary figures and images for: A MAM7 Peptide-Based Inhibitor of Staphylococcus aureus Adhesion Does Not Interfere with In Vitro Host Cell Function
Source: PLoS One. 2013 Nov 12;8(11):e81216. doi: 10.1371/journal.pone.0081216 (PMC3827224; doi:10.1371/journal.pone.0081216)

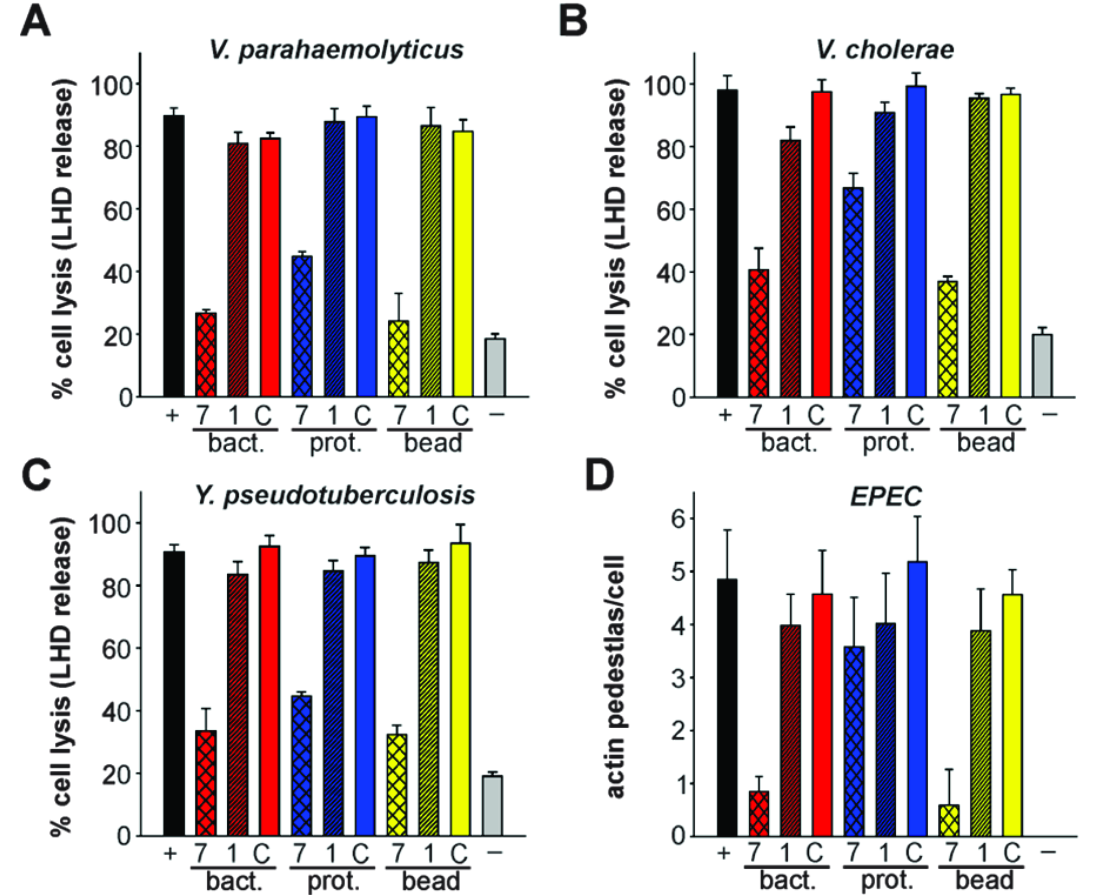

Supplement: Figure S1 — Comparison of different MAM7-based inhibitors for their efficacy against enteric pathogens. Following treatment with MAM-based adhesion inhibitors, Hela cells were infected with V. parahaemolyticus (A), V. cholera (B), Y. pseudotuberculosis (C) or EPEC (D), as previously described [11]. Inhibitors consisted either of MAM7 peptide (7) or MAM7 mce1 domain (1) expressed on the surface of E. coli BL21 (bact.), purified recombinant protein (prot) or bead-coupled protein (bead). As control, cells were treated with BL21 expressing MAM7ΔN1-44 (not surface exposed), GST-tag only (prot C) or bead-coupled GST-tag (bead C). (+) infected cells without prior treatment; (-) uninfected cells; LDH release was measured and drawn as % cell lysis (normalized to 100% = detergent lysed cells). For EPEC, pedestals per cell were counted as previously described [11]. Results are means ± standard deviation from experiments done in triplicate. (TIF) [file pone.0081216.s001.tif]

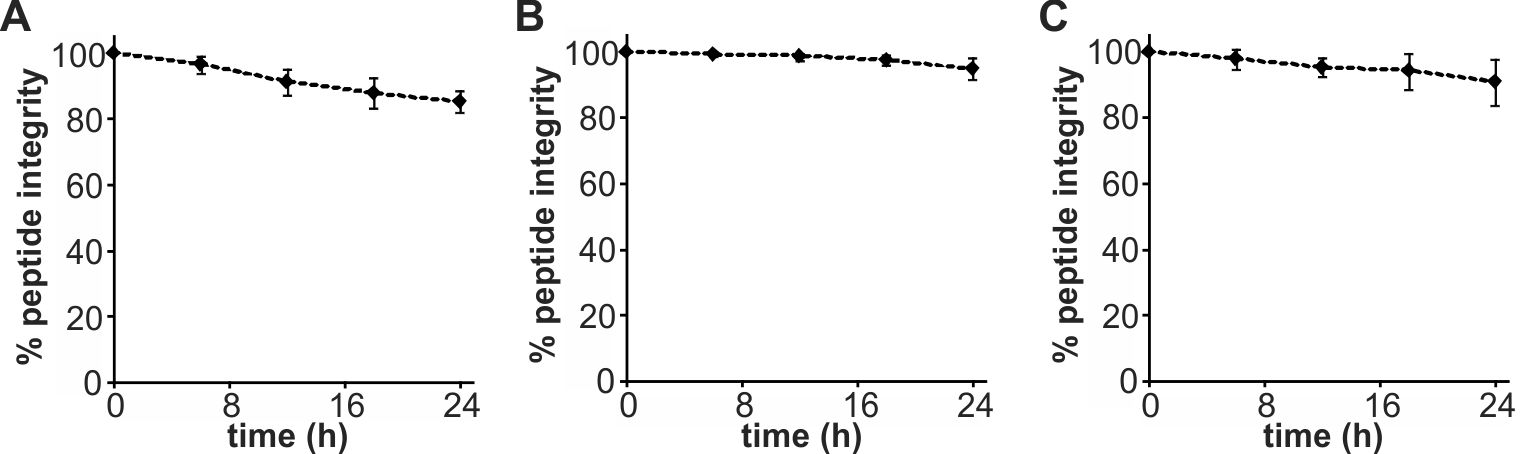

Supplement: Figure S2 — Integrity of anti-adhesion peptide inhibitors over time. The integrity of MAM7, F1 or FnBPA peptides co-incubated with fibroblast monolayers was determined using SDS-PAGE and densitometry. Values are expressed as % integrity and results are means ± standard error from three replicates. (TIF) [file pone.0081216.s002.tif]

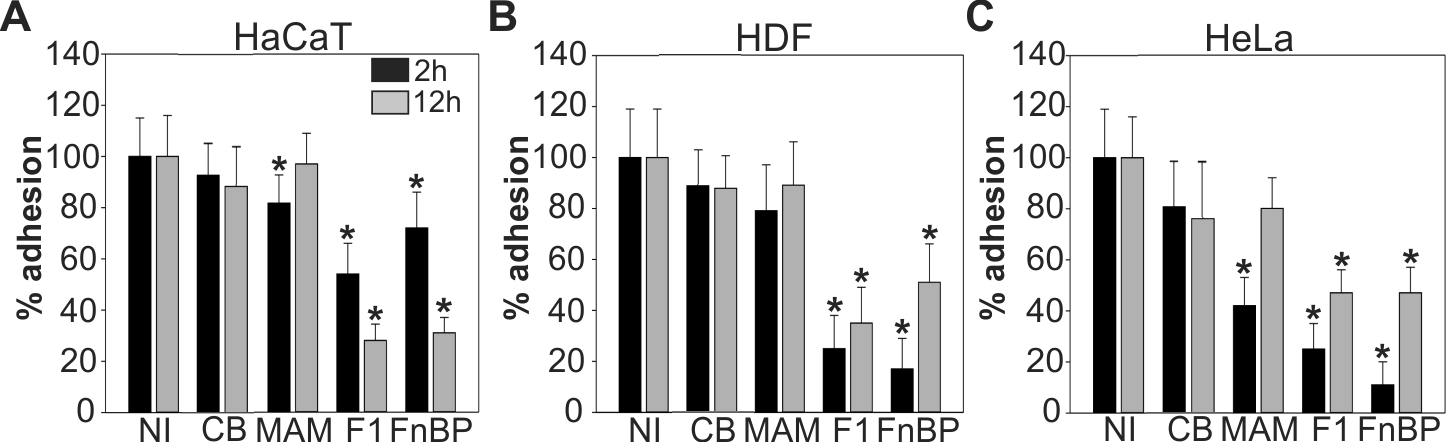

Supplement: Figure S3 — Effect of adhesion inhibitors on host cellular adhesion in the absence of serum. Adhesion of HaCaT (a), HDF (b) or HeLa cells (c) to culture vessels was measured following 2 hours (black) or 12 hours (grey) of incubation in serum-free medium alone (no inhibitor, NI) or medium containing 500 nM bead-immobilized GST (CB), MAM7, F1 or FnBPA peptide. Results are means ± standard deviation from one of a set of three experiments done in triplicate. Values significantly different from the control (p<0.01 according to student t-test) are indicated (*). (TIF) [file pone.0081216.s003.tif]
